# Do Chimpanzees Use Weight to Select Hammer Tools?

**DOI:** 10.1371/journal.pone.0041044

**Published:** 2012-07-18

**Authors:** Cornelia Schrauf, Josep Call, Koki Fuwa, Satoshi Hirata

**Affiliations:** 1 Department of Behavioural Biology, University of Vienna, Vienna, Austria; 2 Department for Developmental and Comparative Psychology, Max Planck Institute for Evolutionary Anthropology, Leipzig, Germany; 3 Great Ape Research Institute, Hayashibara Biochemical Laboratories, Tamano, Okayama, Japan; Yale University, United States of America

## Abstract

The extent to which tool-using animals take into account relevant task parameters is poorly understood. Nut cracking is one of the most complex forms of tool use, the choice of an adequate hammer being a critical aspect in success. Several properties make a hammer suitable for nut cracking, with weight being a key factor in determining the impact of a strike; in general, the greater the weight the fewer strikes required. This study experimentally investigated whether chimpanzees are able to encode the relevance of weight as a property of hammers to crack open nuts. By presenting chimpanzees with three hammers that differed solely in weight, we assessed their ability to relate the weight of the different tools with their effectiveness and thus select the most effective one(s). Our results show that chimpanzees use weight alone in selecting tools to crack open nuts and that experience clearly affects the subjects’ attentiveness to the tool properties that are relevant for the task at hand. Chimpanzees can encode the requirements that a nut-cracking tool should meet (in terms of weight) to be effective.

## Introduction

Wild chimpanzees display a variety of tool-using behaviors. Among these, nut cracking has been considered as one of the most complex forms [1]–[3]. In its most sophisticated variant, a chimpanzee cracks open a nut to access its nutritious kernel by placing the nut on the flat surface of an anvil stone and then hitting the nut with the flat side of a hammer stone [4], [5]. This requires not only the use of two tools (hammer and anvil) but also producing two spatial relations in sequence, one between the nut and the anvil and one between the tool and the nut [6], [7].

Nut-cracking behavior is found in several communities of wild chimpanzees (Guinea: [4], [8], [9]; Ivory Coast: [10], [11], [12]; Liberia: [13], [14]; Sierra Leone: [15]). In East and Central Africa, nut cracking is completely absent [16], [17], apart from a single population of chimpanzees that live east from the Ivory Coast [18]. This is particularly intriguing given that all the necessary elements (nut species, supply of stones, sticks and roots for anvils) are available [19] thus, ecological factors alone cannot explain the absence of this behavior [20]. Furthermore, even within nut-cracking communities, some individuals never acquire the skill [5].

Infant chimpanzees in the Bossou community start to crack open oil-palm nuts at 3.5–6 years of age [5]. In the Taï forest, Ivory Coast, no chimpanzee younger than 5 years has been observed to successfully open coula nuts (*Coula edulis*) [21], [22], even though the younger chimpanzees used the appropriate materials and behavior. Learning nut cracking requires a longer process than other types of simpler tool use [23]. Boesch and Boesch [24] reported that it takes about 4 years of practice until chimpanzees become proficient nut crackers, whereas infant chimpanzees in Bossou need between 3 and 7 years to master the skill [5].

Laboratory studies on the acquisition of nut cracking indicate that not only cognitive abilities are required [25], [14], [26]. A female chimpanzee named Ai who had been very successful in solving computerized experiments failed to learn to use stones as hammers to crack open nuts [27], [26]. The authors argued that Aís failure derived from her lacking the hitting action and her insufficient stone manipulation during this task. Instead of hitting the nut with a stone, Ai pressed the nut with her hand or foot. These behaviors have also been observed in wild chimpanzee infants who have not yet acquired stone tool use [2]. Nonetheless, a few laboratory studies have reported captive chimpanzees successfully learning to crack open nuts using a hammer and anvil [25]: 3 of 5 chimpanzees successfully acquired this behavior; [28]: 1 of 5 chimpanzees learned the skill; [29]: all 5 chimpanzees tested learned to crack open nuts; [26]: 2 of 3 chimpanzees succeeded at this task. These results reinforce the idea that nut cracking is a difficult skill for chimpanzees to acquire and that even extensive training does not guarantee that all individuals will eventually succeed.

The full mastery of nut cracking requires that a chimpanzee attends to the properties of the tools that are relevant for reaching the goal of cracking the nut. Wild chimpanzees have been observed to select appropriate hammers of particular size, shape and material, suggesting that they are able to encode the properties that make a suitable hammer [30], [8], [4]. Boesch and Boesch [12], for instance, found that wild chimpanzees in the Taï forest select hammers and anvils according to the hardness of the nut; for the very hard panda nuts (*Panda oleosa*), chimpanzees exclusively use stone hammers, whereas for the softer coula nuts (*Coula edulis*) they select more wooden hammers. In addition, when only stones are used, bigger, heavier and harder hammers were employed for panda nuts than for coula nuts. Similarly, captive chimpanzees released on an island in Liberia tended to use heavy stones to crack open palm nuts [14], and Bossou chimpanzees selected stones as hammers and anvils based on their size and weight [30], [9].

In nut cracking, hammer weight is a key factor determining the impact of a strike; in general, the greater the weight the fewer strikes are required. Weight is therefore a crucial feature because differences in hammer weight are directly related to the degree of efficiency to crack open nuts. In all the above-mentioned studies, several factors (e.g., material, resistance, friability, shape and weight) affected a hammer’s suitability, some being more important than others. Whether chimpanzees are able to choose the most appropriate hammer based solely on weight is an open question.

Nut-cracking activity is not restricted to chimpanzees. Field observations have shown that capuchin monkeys also use stones as hammers to crack open nuts [7]. In contrast to chimpanzees, who adopt a seated posture to crack nuts and mostly use one hand [5], [22], capuchin monkeys adopt a bipedal posture and raise the stone above their shoulder using both hands [31]. Visalberghi et al. [32] recently investigated whether capuchin monkeys are selective in their choice of hammers in terms of weight to crack nuts. In a series of elegant field experiments, the authors presented wild capuchins with stones differing in functional features (friability and weight). The results were clear: Capuchin monkeys chose, transported, and used the most effective stone to crack open nuts even when the tools were visually identical and weight was the only discriminative feature.

The present study was designed to similarly assess whether captive chimpanzees are able to selectively use tools based solely on weight to crack nuts. In Experiment 1 we presented six chimpanzees with three cuboid-shaped hammers identical in shape, size, material, and color, but differing in weight. Our goal was to assess whether chimpanzees are able to encode the relevance of the tool property weight by relating the weight of the different tools with their effectiveness and showing a preference for the most effective one(s). In Experiment 2, we modified the hammers’ shape and presented spheric hammers. Our aim was to test whether efficiency is affected by a shape change, given that cuboidal tools might constrain accurate handling. In Experiment 3, we altered the weight of the spheric hammers to increase the discrepancy in tool efficiency and to measure how that affected tool selectivity.

## Experiment 1: Materials and Methods

Chimpanzees faced a situation that promoted the selective use of tools: a hard-shelled nut, an anvil, and tools that differed in no other perceivable characteristic than weight (and thus effectiveness). To ensure the perception of the weight differences, the experimenter gave the chimpanzees the three hammers consecutively in their hands before starting the experiment.

### Subjects

Six chimpanzees (*Pan troglodytes versus*) housed at the Great Ape Research Institute (GARI, Okayama, Japan) participated in the study: Loi (male, 13 years 10 months), Zamba (male, 13 years 10 months), Tsubaki (female, 13 years 3 months), Mizuki (female 12 years, 5 months), Misaki (female, 10 years 4 months) and Natsuki (female, 3 years 10 months). All subjects belonged to the same group, with Loi being the alpha male and Zamba the subordinate male. Loi and Zamba were tested individually. Mizuki and Misaki were tested together as well as Tsubaki and her daughter Natsuki because it was impossible to separate them. In these cases a second experimenter distracted one of the chimpanzees while the other participated in the experiment.

At the time of the study, all subjects were familiar with nut cracking and, except Natsuki, they had taken part in a previous nut-cracking experiment [33]. Prior to starting the experiment, each subject was presented once with one of the tools (the lightest) that we used in the experiment later on. This was done for habituation and to help subjects to overcome neophobia.

This research was conducted in accordance with the “Guide for the Care and Use of Laboratory Animals” of Hayashibara Biochemical Laboratories, Inc., and the Weatherall report, “The use of non-human primates in research”. Chimpanzees were housed in semi-natural indoor and outdoor enclosures with regular feedings, daily enrichment and water ad lib. Subjects voluntarily participated in the study and were never food or water deprived. Research was conducted in the observation room. No medical, toxicological or neurobiological research of any kind is conducted at GARI. Research was non-invasive and the research protocols reported in this manuscript were approved by the Animal Welfare and Animal Care Committee and Hayashibara Biochemical Laboratories, Inc. (GARI-090601).

### Apparatus

**Figure 1:**
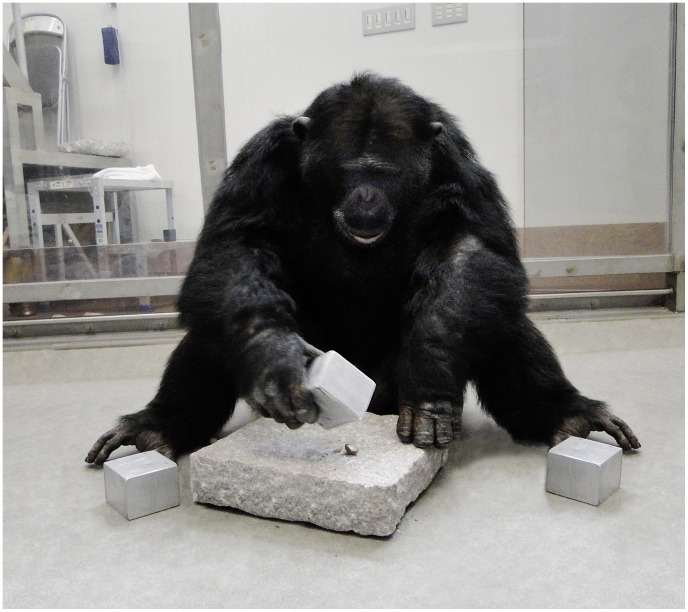
A chimpanzee using a cuboid-shaped hammer to crack open a nut in a pit of the anvil.

The experiment took place in an indoor experimental area (7.6 m^2^, 5 m height) that was connected to the outdoor enclosure. We used Macadamia nuts, which were already well-known to the chimpanzees at the time of testing [33]. Cracking the nutshell required the use of a hammer. Subjects were provided with three visually identical aluminum cuboids (6 cm×8 cm×6 cm) to be used as hammers. The heavy (1200 g) and the mid-weight (600 g) cuboids were filled with lead shot, whereas the light cuboid (300 g) was empty. Transparent silicone paste was mixed with the lead shot to produce a solid and homogenous mass; this prevented any rattling noise during manipulations and evenly distributed the weight inside the tool. A granite stone (30 cm×30 cm and 7 cm high) served as an anvil with five pits (approximately 2 cm of diameter and 0.5–1 cm deep) on its upper surface to place nuts (Figure 1).

### Procedure

The experiment took place from June until September 2009. We conducted one session of six trials per day, for a total of 18 daily sessions (108 trials, i.e., 108 nuts cracked open). After the chimpanzee had entered the experimental room, it sat on a wooden platform directly in front of the stone anvil. Then the experimenter (E, henceforth) entered and sat behind the anvil facing the chimpanzee. Prior to the first trial of a session (to ensure the perception of the differences in weight), E gave the chimpanzee the three hammers consecutively before beginning the experiment. After the chimpanzee had returned the tools, E placed them next to the anvil at a distance of 2–3 cm from each other in front of the subject. The positioning of the tools followed a fixed protocol. Each tool was randomly assigned to one of the three positions (left, right, middle) with the following constraint: a given tool could not be placed consecutively in the same position and, overall, the number of times in which each tool was in one of the three positions was equal at the end of the experiment. A trial started by E putting a macadamia nut in one of the anvil pits; the nut was always placed in the same pit, but the subjects were able to change the nut’s position. Subjects were free to use any of the three provided hammers, meaning that they were allowed to replace the tool first chosen with any other tool during the experiment. Each trial lasted until the chimpanzee had cracked open the nut and started to eat the kernel. Importantly, only after three trials E removed the tools and then gave the chimpanzee the three hammers consecutively to ensure that the subjects experienced the differences in weight. After the chimpanzee had returned the tools, E repositioned the tools using the above protocol and the three remaining trials ensued.

### Data Analysis

Each session was videotaped with a SONY SR-12 camera. We scored from the tapes the identity of the tool used to perform the strike, the number of hits and the time to solution. *Success* of each tool was assessed in terms of the number of strikes and the time required to crack open a nut. For this analysis only those trials in which a single tool was used to open the nut were considered. In addition, we also calculated which hammer type led to success, i.e., the tool that was being used when the nut cracked open. Tool *selectivity* was assessed by tool choice, both overall and first choice as well as switching between tools. Overall tool choice was the frequency with which each tool was chosen, whereas first choice referred to the first tool selected. Switching behavior was measured by calculating the frequency a subject switched from one tool to another tool and the number of times the switch resulted in discarding a lighter tool and selecting a heavier one or vice versa. For the first choice of tools, only the first choice of the first and fourth trial could be considered independent and contributed to the analysis because the tools were not repositioned after every trial. Tool choice and the hammer type were calculated using the data obtained from all six trials of every single session.

We used the Friedman test to assess whether the number of strikes and time to solution differed across tools; pair-wise comparisons between tools were performed with the exact two-tailed Wilcoxon test. We used the Kruskal-Wallis test to assess whether at the individual level the number of strikes and time needed differed between tools; the Mann-Whitney U test was used for the pair-wise comparisons. We used the Friedman test to assess tool selectivity by comparing the values of the three tools both overall and in subjects’ first and last choice. Finally, we used the Chi-square test to assess if a subject selected a particular tool significantly more often than the other tools.

## Experiment 1: Results and Discussion

### Success

#### Number of strikes

**Figure 2:**
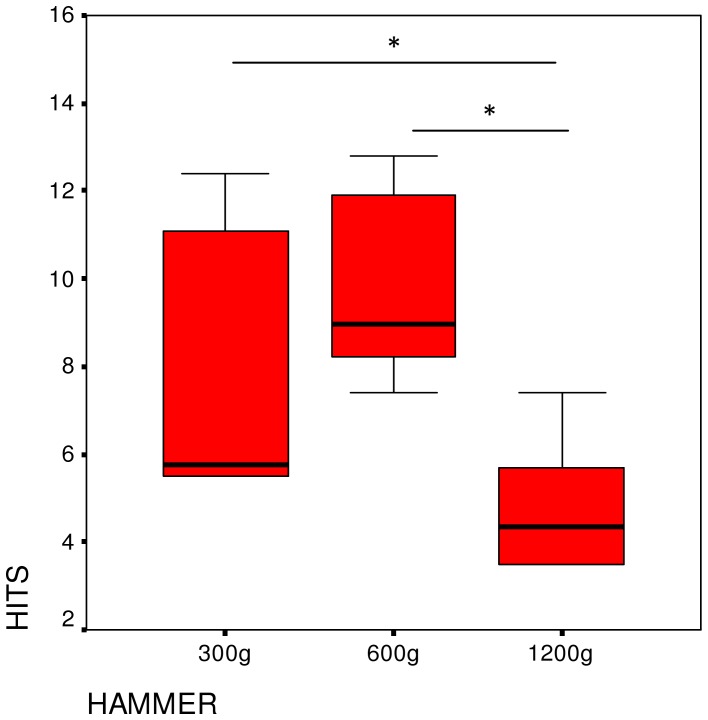
Median number of strikes needed to crack open a nut in Experiment 1. Also shown are the IQR and significance tests.

The number of strikes needed to open a nut and access the kernel differed significantly depending on the hammer (Friedman-test: X^2^ = 10.3, P = 0.006, df = 2) (Figure 2). Usage of the heaviest tool required fewer hits than that of the lightest (Wilcoxon exact test Z = −2.201, P = 0.031) and mid-weight tool (Wilcoxon exact test Z = −2.201, P = 0.031). Although there were no significant differences between the lightest and the mid-weight tool, a trend was seen (Wilcoxon exact test Z = −1.992, P = 0.063). An analysis for each individual showed that the number of strikes needed differed significantly among the three tools for all chimpanzees (Table 1).

**Table 1:** Average number of strikes (±SE) needed to solution for all subjects as a function of hammer weight in Experiment 1.

	Hammer weight					
Subject	300 g	600 g	1200 g	Kruskal-Wallis test: X^2^	P-Values (df = 2)	Pair-wise comparisons
Loi	5.7±0.7	8.5±0.9	3.5±0.3	40.032	0.000	L < M < H
Zamba	5.8±0.7	7.4±1.4	4.6±1	6.654	0.036	L, M, H; M < H
Natsuki	12.4±1.7	11.9±1.5	5.7±0.4	20.047	0.000	L, M < H
Tsubaki	11.1±1.5	12.8±1.9	7.4±0.8	9.044	0.011	L, M < H
Mizuki	5.5±0.5	8.2±1.3	3.5±0.4	17.914	0.000	L, M < H
Misaki	5.5±0.5	9.4±1.5	4.1±1	24.146	0.000	L < M < H

Also shown are the results for the overall significance test and the corresponding pair wise comparisons (“<” denotes a significant difference between hammers).

#### Time to solution

**Figure 3:**
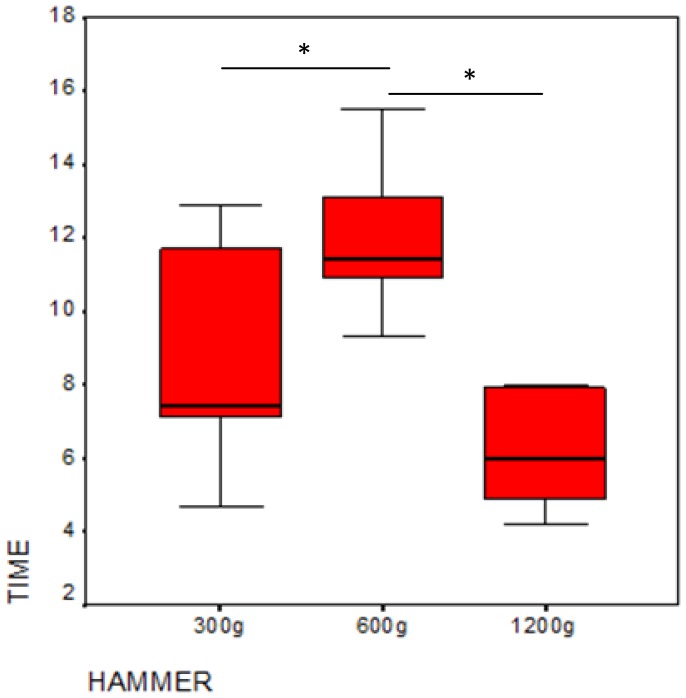
Median time needed to crack open a nut as a function of hammer weight in Experiment 1.

We found significant differences among the three tools in the time needed to crack open the nut (Friedman-test: X^2^ = 9.3, P = 0.009, df = 2) (Figure 3). Although there was no difference in time needed between the heaviest and lightest hammers (Wilcoxon exact test Z = −1.577, P = 0.156), usage of the heaviest hammer required less time than the mid-weight hammer (Wilcoxon exact test Z = −2.207, P = 0.031). When employing the lightest tool, less time was needed than with the mid-weight tool (Wilcoxon exact test Z = −2.201, P = 0.031). Analyzing the data at the individual level revealed that the time needed differed significantly depending on the hammer for Loi, Natsuki, Tsubaki, Mizuki and Misaki (Table 2). In contrast, no such differences were evident for Zamba.

**Table 2:** Average time (±SE) to solution for all subjects as a function of hammer weight.

	Hammer weight					
Subject	300 g	600 g	1200 g	Kruskal-Wallis test: X^2^	P-Values (df = 2)	Pair-wise comparisons
Loi	7.4±1.5	10.9±1.6	4.2±0.4	33.518	0.000	L < M< H
Zamba	7.5±0.9	13.1±4	8±1.6	0.672	0.715	L, M, H
Natsuki	11.7±2.4	11.8±1.8	6.7±0.5	6.429	0.040	L, M < H
Tsubaki	12.9±2.7	15.5±3.3	7.9±1	6.509	0.039	L, M, H; M < H
Mizuki	4.7±0.3	9.3±2.5	4.9±0.9	8.819	0.012	L, M < H
Misaki	7.1±0.9	11.1±2	5.2±1.1	20.046	0.000	L < M < H

Also shown are the results for the overall significance test and the corresponding pair-wise comparisons (“<” denotes a significant difference between hammers).

#### Hammer type

Overall we found no significant differences in the choice of tools that led to success, i.e., the hammer that cracked the nut (Friedman-test: X^2^ = 1.182, P = 0.554, df = 2). When analyzing subjects’ individual choices, we found significant differences in tool choice that led to success only for Loi (X^2^ = 37.5, P<0.001, df = 2, N1 = 21, N2 = 21, N3 = 66) and Zamba (X^2^ = 6, P = 0.05, df = 2, N1 = 48, N2 = 30, N3 = 30). The remaining subjects showed no hammer preferences (Chi-square tests: Natsuki: X^2^ = 0.5, P = 0.779, df = 2, N1 = 33, N2 = 39, N3 = 36; Tsubaki: X^2^ = 3.722, P = 0.155, df = 2, N1 = 34, N2 = 29, N3 = 45; Mizuki: X^2^ = 0.389,P = 0.823, df = 2, N1 = 39, N2 = 34, N3 = 35; Misaki: X^2^ = 3.556, P = 0.169,df = 2; N1 = 44, N2 = 36, N3 = 28).

### Tool Selectivity

#### First choice

Subjects as a group did not show any preference for a certain tool (Friedman-test: X^2^ = 2.8, P = 0.247, df = 2). Furthermore, none of the subjects’ observed individual choices diverged from expected levels (Chi-square tests: Loi: X^2^ = 0.167, P = 0.92, df = 2, N1 = 11, N2 = 13, N3 = 12; Zamba: X^2^ = 2.167, P = 0.338, df = 2, N1 = 16, N2 = 11, N3 = 9; Natsuki: X^2^ = 0, P = 1, df = 2, N1 = 12, N2 = 12, N3 = 12; Tsubaki: X^2^ = 0.167, P = 0.92, df = 2, N1 = 13, N2 = 12, N3 = 11; Mizuki: X^2^ = 0.167, P = 0.92, df = 2; N1 = 12, N2 = 13, N3 = 11; Misaki: X^2^ = 1.167, P = 0.558, df = 2, N1 = 15, N2 = 10, N3 = 11).

#### Overall choice

Subjects as a group showed no preference for a particular tool (Friedman-test: X^2^ = 0.333, P = 0.846, df = 2). Analyzing the data at the individual level revealed that Loi displayed a clear preference for the heaviest hammer (X^2^ = 14.057, P = 0.001, df = 2, N1 = 45, N2 = 49, N3 = 82) while Zamba favored the lightest hammer (X^2^ = 6.615, P = 0.037, df = 2, N1 = 52, N2 = 34, N3 = 31). The remaining subjects showed no preference for a particular hammer (Chi-square tests: Natsuki: X^2^ = 0.349, P = 0.840, df = 2, N1 = 34, N2 = 39, N3 = 36; Tsubaki: X^2^ = 2.864, P = 0.239, df = 2, N1 = 39, N2 = 32, N3 = 47; Mizuki: X^2^ = 0.125, P = 0.939, df = 2, N1 = 39, N2 = 37, N3 = 36; Misaki: X^2^ = 2.619, P = 0.270, df = 2, N1 = 45, N2 = 37, N3 = 31).

#### Switching between tools

Table 3 presents the frequency of tool switching both prior and after using the hammer. Overall, subjects showed no clear difference between the preference for switching from light to heavy hammers or from heavy to light hammers (Wilcoxon exact test: Z = −1.16, P = 0.34). One individual (Loi), however, showed a tendency for switching from light to heavy hammers rather than vice versa (Binomial test: P = 0.057, N = 71), although this preference was mostly observed after using the hammer.

**Table 3:** Number of total switches observed as a function of hammer weight (L = 300 g, M = 600 g, H = 1200 g) and the direction of switches (L->H = from lighter to a heavier; H->L = from heavier to lighter) in Experiment 1.

	Total switches			after use		before use	
Subject	L	M	H	L->H	H->L	L->H	H->L
LOI	26	28	17	42	26	2	1
ZAMBA	5	4	1	6	3	1	0
NATSUKI	1	0	0	1	0	0	0
TSUBAKI	5	3	2	7	3	0	0
MIZUKI	1	3	1	2	2	1	0
MISAKI	1	2	5	1	4	0	3
TOTAL	39	40	26	59	38	4	4

#### Discussion of Experiment 1

Two chimpanzees, Loi and Zamba, differentiated between three visually identical hammers differing only in weight to crack open a nut. In particular, these two subjects showed a preference for a certain hammer weight in their overall tool choice, and this selectivity emerged from the chimpanzees experiencing the differences in tool effectiveness. Loi preferred the heaviest (and most efficient) hammer, Zamba the lightest. None of our subjects showed an initial preference for a certain hammer in their first choices. This absence excludes the possibility that our subjects had used any inadvertent cueing (e.g., scratches on the tools’ surface) instead of weight. Subjects did not remain with the tool first chosen but switched from striking with one tool to striking with another tool. Switching behavior, which further indicates that the tools were valued differently, was most frequently recorded for Loi, who performed more switches from a lighter to a heavier tool than vice versa. Loi also showed a preference for a certain hammer weight in his choice of tools that led to success. In particular, Loi kept on using the heaviest hammer far more often than the mid-weight or lightest hammer until the nutshell cracked.

Note here that Loi, who apparently outperformed all the other subjects, had substantially greater experience in nut cracking, having been trained to crack nuts by humans in the past. The other chimpanzees had learned this behavior through social learning sessions, Loi being the model [29]. It is plausible that Loi’s extensive experience allowed him to be more attentive to the relevant tool properties. In fact, a study in human craftsmen has shown that skilled subjects take better advantage of the tool properties [34]. Thus, in our study, although all subjects might have perceived the weight differences, the deeper relationship between weight and efficiency was accessible only to Loi.

It is nevertheless surprising that tool selectivity did not emerge in all subjects, given that the hammers differed significantly in effectiveness. One explanation is that all tools were functional to some extent, i.e. in the sense that with enough hits it was possible to crack the nutshell with all three tools. Using a lighter tool was therefore not strictly a mistake, it was merely less efficient. Consequently, subjects might have used substandard tools because hammers have to be either functional or non functional or the difference in efficiency has to be larger so that chimpanzees benefit more from choosing a particular tool.

Finally, hammer choice might not only be based on weight but also be constrained by how easily a hammer can be held and accurately handled, which is size, weight and shape dependent. Given that tool effectiveness in our study did not correlate linearly with weight (i.e., using the lightest tool required fewer hits and time than the mid-weight tool), this assumption is very likely. Perhaps the cuboidal shape of our provided tools is problematic for accurate handling, as angles must be taken into account when matching the hammer surface with the nut and anvil.

## Experiment 2

In Experiment 2 we modified the hammerś shape and replaced the cuboid with spherical hammers. Our aim was to measure whether efficiency is affected by altered shape and consequentially if that shape reinforces tool selectivity.

## Experiment 2: Materials and Methods

### Participants

All the subjects who had participated in Experiment 1 took part in Experiment 2.

### Apparatus

**Figure 4:**
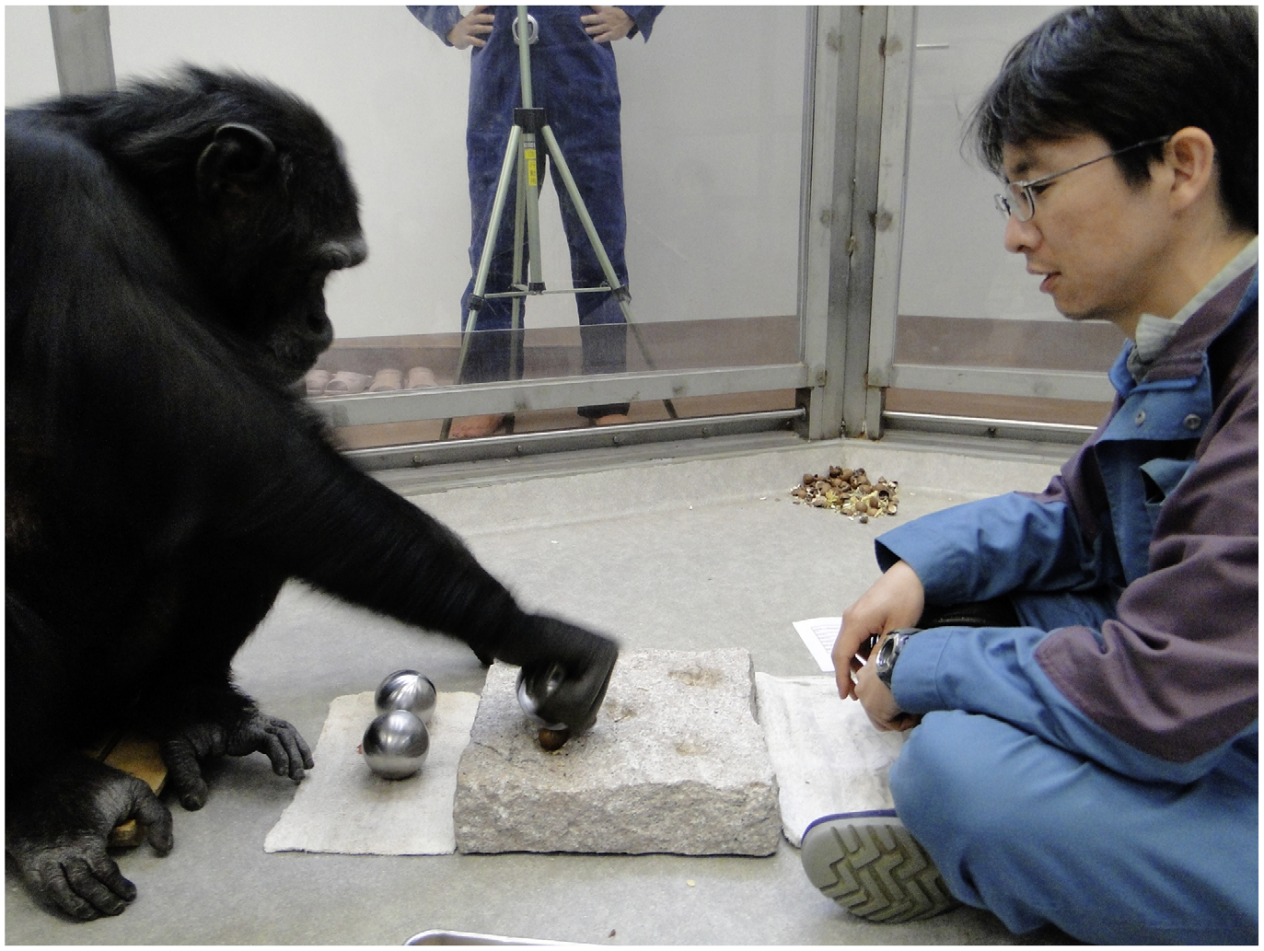
A chimpanzee using a spherical hammer to crack open a nut in a pit of the anvil.

Three visually identical spherical aluminum hammers were presented as tools. The hammers were 7 cm in diameter and weighed 300 g, 600 g and 1200 g, respectively. The same anvil and nut species were used as in Experiment 1 (Figure 4).

### Procedure

The experiment took place from March until mid-May 2010. We conducted one session of six trials per day, for a total of 6 daily sessions (36 trials, i.e., 36 nuts cracked open). Except for the different hammers and the number of sessions administered, the experimental procedure and data analyses were the same as in Experiment 1.

## Experiment 2: Results and Discussion

### Success

#### Number of strikes

**Figure 5:**
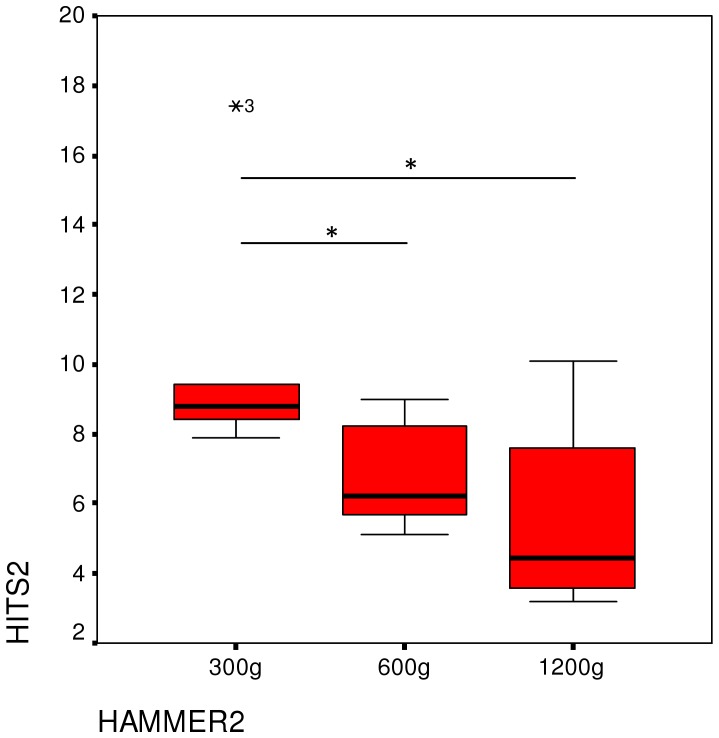
Median number of strikes needed to crack a nut in Experiment 2. Also shown are the IQR and significance tests.

The number of strikes needed to open a nut and access the kernel differed significantly depending on the hammer (Friedman-test: X^2^ = 10.3, P = 0.006, df = 2) (Figure 5). There was a significant difference in the number of hits needed to crack open a nut between the heaviest and lightest hammers (Wilcoxon exact test Z = −2.201, P = 0.031). Using the heaviest tool, however, did not require significantly fewer hits than the mid-weight tool (Wilcoxon exact test Z = −1.472, P = 0.188). In contrast to Experiment 1, employing the lightest hammer necessitated more hits than the mid-weight hammer (Wilcoxon exact test Z = −2.201, P = 0.031). Analyzing the data on an individual level (Table 4) showed that strike number to crack a nut differed significantly among the three tools for all subjects except Tsubaki.

**Table 4:** Average number of strikes (±SE) needed to solution for all subjects as a function of hammer weight in Experiment 2.

	Hammer weight					
Subject	300 g	600 g	1200 g	Kruskal-Wallis test: X^2^	P-Value (df = 2)	Pair-wise comparisons
Loi	8.8±0.9	5.1±0.5	4.3±0.6	12.604	0.002	L < M, H
Zamba	7.9±0.7	5.8±0.6	3.6±0.6	16.429	0.000	L < M < H
Natsuki	17.4±2.9	9±1.5	10.1±1.6	7.144	0.028	L < M, H
Tsubaki	8.8±1.2	8.2±0.9	7.6±1	0.909	0.635	L, M, H
Mizuki	8.4±1	5.7±1	4.6±0.7	7.644	0.022	L, M, H; L < H
Misaki	9.4±1.1	6.6±1	3.2±0.5	19.223	0.000	L, M < H

Also shown are the results for the overall significance test and the corresponding pair-wise comparisons (“<” denotes a significant difference between hammers).

#### Time to solution

**Figure 6:**
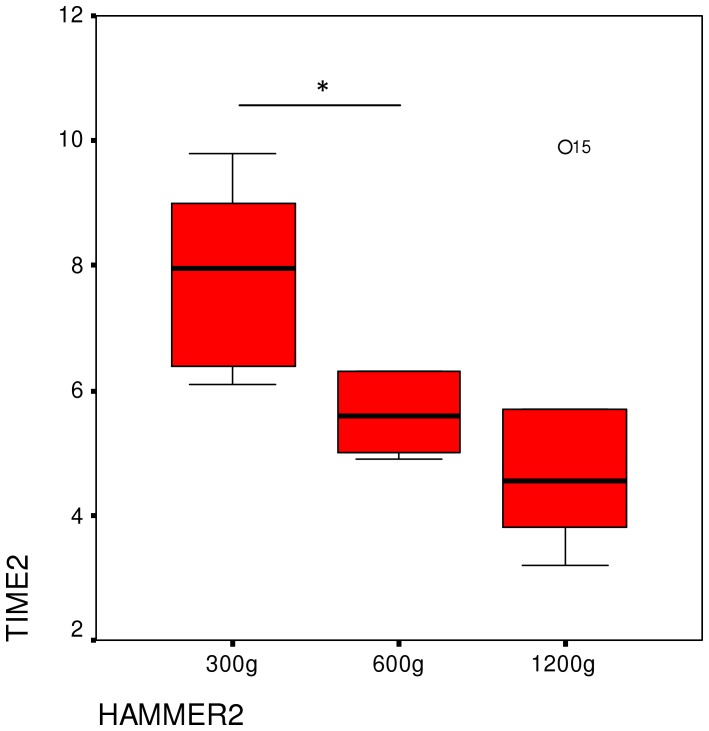
Median time needed to crack open a nut as a function of hammer weight in Experiment 2 .

The time needed differed significantly among the three tools (Friedman-test: X^2^ = 7, P = 0.030, df = 2) (Figure 6). When employing the lightest hammer, more time was needed than with the mid-weight hammer (Wilcoxon exact test Z = −2.207, P = 0.031). The use of the heaviest tool required less time than the lightest tool, although this finding did not reach significance level (Wilcoxon exact test Z = −1.997, P = 0.063). Similarly, time usage did not differ between the heaviest and mid-weight tool (Wilcoxon exact test Z = −0.946, P = 0.406). At the individual level, the time needed to crack the nut differed significantly among the three tools for Loi, Zamba and Misaki (Table 5). No differences were found for Natsuki, Tsubaki and Mizuki.

**Table 5:** Average time (±SE) to solution for all subjects as a function of hammer weight.

	Hammer weight					
Subject	300 g	600 g	1200 g	Kruskal-Wallis test: X^2^	P-Values (df = 2)	Pair-wise comparisons
Loi	7.5±1	5.4±0.4	4.8±0.6	8.356	0.015	L < M, H
Zamba	6.1±0.4	4.9±0.5	3.8±0.5	14.097	0.001	L, M < H
Natsuki	9±1.4	6.3±0.9	9.9±2.4	2.763	0.251	L, M, H
Tsubaki	8.4±2	6.3±0.6	5.7±0.6	1.740	0.419	L, M, H
Mizuki	6.4±0.6	5±0.8	4.3±0.5	5.892	0.053	L, M, H; L < H
Misaki	9.8±1.8	5.8±0.8	3.2±0.3	15.516	0.000	L, M < H

Also shown are the results for the overall significance test and the corresponding pair-wise comparisons (“<” denotes a significant difference between hammers).

#### Hammer type

The frequency in the choice of tools that led to success, i.e., the hammer that cracked the nut, differed significantly at the group level (Friedman-test: X^2^ = 7.636, P = 0.022, df = 2). At the individual level, however, the difference was not significant (Chi-square tests: Loi: X^2^ = 4.5, P = 0.105, df = 2, N1 = 6, N2 = 15, N3 = 15; Zamba: X^2^ = 1.5, P = 0.472, df = 2, N1 = 12, N2 = 9, N3 = 15; Natsuki: X^2^ = 1.5, P = 0.472, df = 2, N1 = 9, N2 = 12, N3 = 15; Tsubaki: X^2^ = 4.5, P = 0.105, df = 2, N1 = 6, N2 = 15, N3 = 15; Mizuki: X^2^ = 3.167, P = 0.205, df = 2, N1 = 9, N2 = 10, N3 = 17; Misaki: X^2^ = 1.167, P = 0.558,df = 2, N1 = 11, N2 = 10, N3 = 15).

### Tool Selectivity

#### First choice

As a group subjectś first choice behavior did not deviate significantly from chance (Friedman-test: X^2^ = 5.143, P = 0.076, df = 2). A statistical analysis at the individual level was not possible due to the small sample size: Loi (300 g N = 2; 600 g N = 5; 1200 g N = 5), Zamba (300 g N = 4; 600 g N = 3; 1200 g = 5), Natsuki (300 g N = 4; 600 g N = 4; 1200 g N = 4), Tsubaki (300 g N = 2; 600 g N = 5; 1200 g N = 5), Mizuki (300 g N = 3; 600 g N = 4; 1200 g N = 5) and Misaki (300 g N = 4; 600 g N = 4; 1200 g N = 4).

#### Overall choice

Subjects as a group showed a preference for the heaviest hammer (Friedman-test: X^2^ = 7.636, P = 0.022, df = 2). The heaviest tool was chosen significantly more often than the lightest tool (Wilcoxon test Z = −2.214, P = 0.027) and more often than the mid-weight tool, although the latter finding did not reach significance level (Wilcoxon test Z = −1.826, P = 0.068). The frequency with which the lightest and the mid-weight tool were chosen also did not deviate from chance level (Wilcoxon test Z = −1.054, P = 0.292).

On an individual level, the subjects choice of tools did not differ significantly (Chi-square tests: Loi: X^2^ = 4.5, P = 0.105, df = 2, N1 = 6, N2 = 15, N3 = 15; Zamba: X^2^ = 1.5, P = 0.472, df = 2, N1 = 12, N2 = 9, N3 = 15; Natsuki: X^2^ = 1, P = 0.607, df = 2, N1 = 10, N2 = 13, N3 = 15; Tsubaki: X^2^ = 4.5, P = 0.105, df = 2, N1 = 6, N2 = 15, N3 = 15; Mizuki: X^2^ = 3.167, P = 0.205, df = 2, N1 = 9, N2 = 10, N3 = 17; Misaki: X^2^ = 1.027, P = 0.598, df = 2, N1 = 12, N2 = 10, N3 = 15).

#### Switching between tools

Only Natsuki and Misaki switched tools, but they did so infrequently (Table 6). Switches occurred only after subjects had already performed several strikes with the initial tool.

**Table 6:** Number of total switches observed as a function of hammer weight (L = 300 g, M = 600 g, H = 1200 g) and the direction of switches (L->H = from lighter to a heavier; H->L = from heavier to lighter) in Experiment 2.

	Switches			after use	
Subject	L	M	H	L->H	H->L
NATSUKI	1	1	0	1	1
MISAKI	1	0	0	1	0
TOTAL	2	1	0	2	1

#### Discussion of Experiment 2

Compared to Experiment 1, subjects as a group developed a preference for the heaviest (and most efficient) hammer, although that preference was not apparent in chimpanzees’ first choices. The fact that they kept on using the heaviest hammer significantly more often than the lightest hammer until the nutshell cracked, also indicates their heightened sensitivity to hammer weight. This result supports the data on hammer efficiency because heavier hammers required fewer hits and less time. In fact, unlike in Experiment 1, tool effectiveness in Experiment 2 did correlate linearly with weight (i.e., using a lighter tool required more hits and time than a heavier tool). Nonetheless, attributing this difference solely to hammer shape would be premature because subjects also had more nut-cracking experience in Experiment 2 than Experiment 1.

## Experiment 3

In Experiment 3 we modified the weight of the spheric hammers to increase the discrepancy in tool efficiency and measure how that affected tool selectivity.

## Experiment 3: Materials and Methods

### Participants

All the subjects who had participated in Experiment 2 took part in Experiment 3.

### Apparatus

Three visually identical spherical aluminum hammers were presented as tools. The hammers were 7 cm in diameter and weighed 200 g, 800 g, and 1400 g, respectively. The same anvil and nut species was used as in Experiment 2.

### Procedure

The experiment took place from mid-May until October 2010. We conducted one session of six trials per day, for a total of 12 daily sessions (72 trials, i.e., 72 nuts cracked open). Except for the different hammers and the number of sessions administered, the experimental procedure was the same as in Experiment 1. Analyses were also the same as in previous experiments.

## Experiment 3: Results and Discussion

### Success

#### Number of strikes

**Figure 7:**
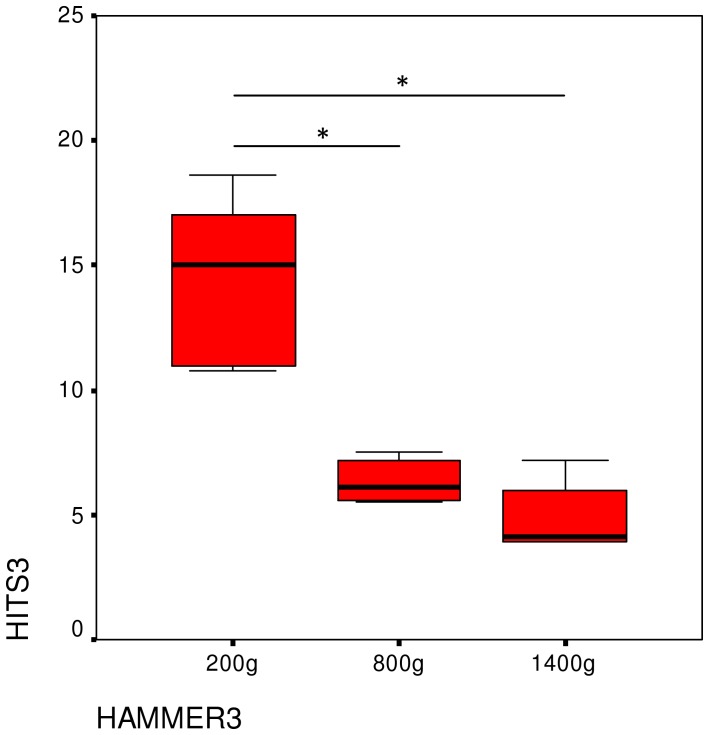
Median number of strikes needed to crack open a nut in Experiment 3. Also shown are the IQR and significance tests.

The number of necessary strikes differed significantly depending on the hammer (Friedman-test: X^2^ = 11.57, P = 0.003, df = 2) (Figure 7). Usage of the heaviest tool required fewer hits than that of the lightest tool (Wilcoxon exact test Z = −2.201, P = 0.031). Although there was no significant difference in strike number between heaviest and mid-weight hammers, a trend was seen (Wilcoxon exact test Z = −2.023, P = 0.063). The lightest hammer required more strikes than the mid-weight one (Wilcoxon exact test Z = −2.201, P = 0.031). On an individual level, the number of necessary strikes differed significantly among the three tools for all subjects except for Tsubaki (Table 7).

**Table 7:** Average number of strikes (±SE) needed to solution for all subjects as a function of hammer weight in Experiment 3.

	Hammer weight					
Subject	200 g	800 g	1400 g	Kruskal-Wallis test: X^2^	P-Values (df = 2)	Pair-wise comparisons
Loi	11.0±1.2	6.0±0.5	3.9±0.3	22.774	0.000	L < M < H
Zamba	14.0±2.0	5.6±0.6	3.9±0.4	27.474	0.000	L < M < H
Natsuki	18.6±2.6	7.5±0.8	6.0±0.7	31.747	0.000	L < M, H
Tsubaki	17±4.8	7.2±0.5	7.2±0.9	5.862	0.053	L < M, H
Mizuki	10.8±2.2	6.3±0.8	4.1±0.4	13.267	0.001	L < M < H
Misaki	16±3.8	5.5±0.5	4.1±0.3	15.120	0.001	L < M < H

Also shown are the results for the overall significance test and the corresponding pair-wise comparisons (“<” denotes a significant difference between hammers).

#### Time to solution

**Figure 8:**
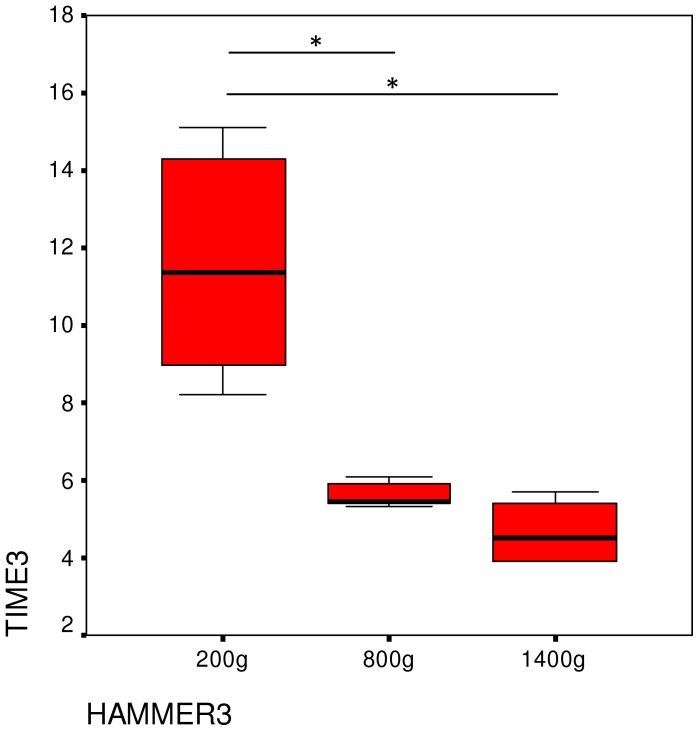
Median time needed to crack open a nut as a function of hammer weight in Experiment 3.

The time needed to crack a nut differed significantly between the three tools (Friedman-test: X^2^ = 10.3, P = 0.006, df = 2) (Figure 8). As with the number of strikes, using the heaviest hammer required less time than the lightest one (Wilcoxon exact test Z = −2.201, P = 0.031) but there was no difference between the heaviest and the mid-weight tool (Wilcoxon exact test Z = −1.782, P = 0.094). Usage of the lightest hammer required more time than of the mid-weight one (Wilcoxon exact test Z = −2.201, P = 0.031). At the individual level, the time needed to crack a nut differed significantly among the three tools for Loi, Zamba, Natsuki and Mizuki (Table 8), but not for Tsubaki and Misaki.

**Table 8:** Average time (±SE) to solution for all subjects as a function of hammer weight.

	Hammer weight					
Subject	200 g	800 g	1400 g	Kruskal-Wallis test: X^2^	P-Value (df = 2)	Pair-wise comparisons
Loi	9±1	6.1±0.4	4.4±0.3	16.727	0.000	L < M < H
Zamba	14.3±3.4	5.9±0.9	3.9±0.4	24.090	0.000	L < M < H
Natsuki	11.3±1.3	5.5±0.4	5.4±0.4	21.402	0.000	L < M, H
Tsubaki	11.4±3.3	5.4±0.3	5.7±0.6	5.234	0.073	L < M, H
Mizuki	8.2±2	5.4±0.6	3.9±0.3	7.635	0.022	L, M, H; L < H
Misaki	15.1±3.6	5.3±0.4	4.6±0.3	13.045	0.001	L < M, H

Also shown are the results for the overall significance test and the corresponding pair-wise comparisons (“<” denotes a significant difference between hammers).

#### Hammer type

The frequency in the choice of tools that led to success, i.e., the hammer that cracked the nut, differed significantly at group level (Friedman-test: X^2^ = 7.636, P = 0.022, df = 2). When analyzing subjectś individual choices, the choice of tools differed significantly for all subjects (Chi-square tests: Loi: X^2^ = 21, P = 0.000, df = 2, N1 = 6, N2 = 36, N3 = 30; Zamba: X^2^ = 9.08, P = 0.011, df = 2, N1 = 12, N2 = 29, N3 = 31; Tsubaki: X^2^ = 25.75, P = 0.000, df = 2, N1 = 4, N2 = 37, N3 = 31; Mizuki: X^2^ = 19, P = 0.000, df = 2, N1 = 8, N2 = 26, N3 = 38; Misaki: X^2^ = 21, P = 0.000, df = 2, N1 = 6, N2 = 30, N3 = 36). The exception was Natsuki, who showed no hammer preference (X^2^ = 0, P = 1, df = 2 N1 = 24, N2 = 24, N3 = 24).

### Tool Selectivity

#### First choice

As a group, subjectś first choice behavior did not deviate significantly from chance (Friedman-test: X^2^ = 2, P = 0.368, df = 2). Furthermore, none of the subjects’ observed first choices diverged from expected (Chi-square tests: Loi: X^2^ = 0.250, P = 0.882, df = 2, N1 = 7, N2 = 8, N3 = 9; Zamba: X^2^ = 0.000, P = 1, df = 2, N1 = 8, N2 = 8, N3 = 8; Natsuki: X^2^ = 0.000, P = 1, df = 2, N1 = 8, N2 = 8, N3 = 8; Tsubaki: X^2^ = 0.250, P = 0.882, df = 2, N1 = 8, N2 = 9, N3 = 7; Mizuki: X^2^ = 0.250, P = 0.882, df = 2, N1 = 7, N2 = 8, N3 = 9 and Misaki: X^2^ = 0.000, P = 1, df = 2, N1 = 8, N2 = 8, N3 = 8).

#### Overall choice

Subjects as a group preferred the heaviest hammer (Friedman-test: X^2^ = 7.6, P = 0.022, df = 2). It was chosen significantly more often than the lightest one (Wilcoxon test Z = −2.023, P = 0.043). No difference in the frequency of choice was found between the heaviest and the mid-weight tool (Wilcoxon test Z = −0.412, P = 0.680). The lightest tool was chosen significantly less often than the mid-weight one (Wilcoxon test Z = −2.032, P = 0.042).

At an individual level, a significant difference was detected in Loi’s, Mizuki’s, Misaki’s and Tsubakís choice of tools (Chi-square tests: Loi: X^2^ = 12, P = 0.002, df = 2, N1 = 12, N2 = 36, N3 = 30; Mizuki: X^2^ = 11.077, P = 0.004, df = 2, N1 = 14, N2 = 26, N3 = 38; Misaki: X^2^ = 9.7, P = 0.008, df = 2, N1 = 14, N2 = 30, N3 = 36 and Tsubaki: X^2^ = 15.462, P<0.001, df = 2, N1 = 10, N2 = 37, N3 = 31). No differences were found for Zamba and Natsuki (Chi-square tests: Zamba: X^2^ = 4.468, P = 0.107, df = 2, N1 = 17, N2 = 29, N3 = 31; Natsuki: X^2^ = 0.000, P = 1, df = 2, N1 = 24, N2 = 24, N3 = 24).

#### Switching between tools

Table 9 presents the frequency of tool switching both prior and after using the hammer. Subjects showed a preference for switching more from light to heavy hammers than from heavy to light ones, although the differences were not significant (Wilcoxon exact test: Z = −2.023, P = 0.063).

**Table 9:** Number of total switches observed as a function of hammer weight (L = 300 g, M = 600 g, H = 1200 g) and the direction of switches (L-> H = from lighter to heavier; H->L = from heavier to lighter) in Experiment 3.

	Total switches			after use		before use	
Subject	L	M	H	L->H	H->L	L->H	H->L
LOI	5	0	1	4	0	1	1
ZAMBA	5	0	0	5	0	0	0
NATSUKI	0	0	0	0	0	0	0
TSUBAKI	10	1	0	6	0	4	1
MIZUKI	7	0	0	6	0	1	0
MISAKI	10	1	1	7	0	4	1
TOTAL	37	2	2	28	0	10	3

#### Discussion of Experiment 3

Subjects as a group preferred the heaviest (and most efficient) hammer. Although we presented new weights, selectivity quickly emerged in most subjects after experiencing the differences in tool effectiveness. Besides Natsuki, the infant, all subjects chose the lightest hammer less often than the heavier ones. This behavior is not surprising given that using the lightest hammer required the most number of hits and time to success. Thus, the discrepancy in tool efficiency was perceived so that subjects benefited more from choosing a particular tool. In fact, most of the subjects started to avoid the lightest tool: they switched from the lightest to a heavier tool in 28 instances. The opposite switch never occurred; importantly, in several instances, subjects switched from the lightest tool before using it, indicating that subjects switched the moment they lifted it and experienced its weight. This strongly suggests that switching was not determined by a failure to crack the nut. Rather, switching was based on anticipating the outcome of the used hammer. Tool selectivity was further evident in the choice of tools that led to success, i.e. the hammer that cracked the nut. In particular, all subjects, besides Natsuki, kept on using the heaviest and mid-weight hammer far more often than the lightest hammer until the nutshell cracked open. Interestingly, the chimpanzees clearly avoided using the lightest and least efficient tool, but did not differ in their choice between the mid-weight and heaviest tool. Given that the difference in effectiveness between the mid-weight and the heaviest hammer was small (especially compared with the lightest hammer) this finding is not surprising, as subjects might not have really benefited from preferring one of these tools. In summary, our results show that 1) not only does hammer weight determine tool efficiency, but a combination of weight, shape and size because this determines accurate handling and 2) chimpanzees are selective in hammer choice (regarding weight) only when they really benefit from choosing a particular tool.

## Discussion

Chimpanzees preferred to use heavier hammers that required fewer hits and less time to crack open nuts. Since all hammers shared the same visual features in terms of size and appearance, hammers were selected according to their relative effectiveness based on their weight. In particular, the choice of a “good” hammer necessitated attributing a specific function to weight as a tool property, namely that higher weight increased the efficiency of a single strike. This extends and refines the results of previous studies, obtained from wild chimpanzees, in which heavier hammers were preferred over lighter ones [30], [12], [9]. As the hammers in all these studies differed in several characteristics (size, material, weight), the question whether chimpanzees choices were based on weight remained unanswered. Our results provide conclusive evidence that chimpanzees use weight alone in selecting tools to crack open nuts.

Experience seemed to play a major role in determining performance, as our most proficient subject, Loi, outperformed the other chimpanzees from the early beginning of the experiment. Already in Experiment 1, Loi clearly made discriminative use of the available tools, using weight to select an appropriate hammer. He showed a preference for the heaviest (and most efficient) hammer and switched twice as much from a lighter to a heavier tool than vice versa. Loi’s prior experience in nut cracking could explain his early apprehension of weight as having a relevant function for nut-cracking tools (specifically, that weight is directly related to the effort and time required).

A study on the ability of captive capuchin monkeys to select hammers according to weight and effectiveness [35] revealed that selectivity for the heaviest hammer emerged very soon in the most proficient subject, Pepe. The authors argued that Pepe’s prior experience with tool-mediated nut cracking could account for this rapidity. Furthermore, when Visalberghi et al. [32] provided semi-free ranging capuchin monkeys with stone tools of different effectiveness to crack open nuts, these wild capuchins outperformed captive capuchins tested in a similar task [7], [35]. The wild capuchins’ superior performance was attributed to a lifelong experience with a variety of nuts and stones and cracking open nuts on a daily basis [7]. This experience allowed subjects to recognize the properties that made a hammer tool effective.

Nevertheless, it is remarkable that most of the subjects in Experiment 1, who showed no discriminative use of the presented tools, started being selective within a few trials in Experiment 2. By the end of Experiment 3, their performance was even comparable to Loi’s, our most experienced subject. It is conceivable that the improved performance can be attributed to the changes in hammer shape and weight in Experiments 2 and 3. However, it is also possible that this preference developed as a result of being confronted with the task multiple times over the course of the study.

Hammer shape may have been another potential contributing factor to the observed increase in selectivity. Compared to the cuboid-shaped hammers, spherically-shaped ones might have allowed subjects to better grab and handle them when striking the nut. Fragaszy et al. [36] stated that the efficiency in capuchin monkeys’ nut-cracking activity depends on the subjects’ control of the stone and the angle of its impact on the nut. This could also be the case for our chimpanzees, especially given our own observations that the cuboidal hammers in Experiment 1 sometimes caused difficulties for the chimpanzees when trying to contact the hammer’s surface with the nut. As the impact of the strike depends on the amount of contact between the hammer and the nut [33] subjects sometimes had to reposition the tool in their hand when they applied insufficient force while striking the nut, which required taking the angles of the hammer into account. Potentially, the important role of weight was masked by the difficulties subjects experienced in trying to accurately handle the tools. Given that tool effectiveness in Experiment 1 did not correlate linearly with weight (i.e. the lightest tool required fewer hits and less time than the mid-weight tool), this assumption is very likely.

Experience alone, however, is not the whole explanation for proficient selection of appropriate hammers. Natsuki, the only infant in our study sample, never improved her performance over the course of the experiments, even though she received the same experience as most of the chimpanzees in the study. Even in Experiment 3, where the differences in tool efficiency differed drastically, Natsuki still selected all three tools the same number of times and never switched tools. This fits the observations in wild juvenile chimpanzees, who sometimes use hammers that are too light to crack open a nut, whereas adults always use heavy tools [37]. Taken together, the amount of experience in nut-cracking activity is apparently a strong predictor for the chimpanzees’ ability to choose a tool, but maturational aspects also play an important role in determining tool selectivity and proficiency.

Capuchin monkeys gained information about the weight of the stones by moving, lifting or tapping them [32] before making their first selection of a stone to be used as hammer. Moreover, they searched for the critical feature (weight) when other cues were identical or contradicted the critical feature. Brosnan [38] interpreted these findings as evidence that capuchins had a true understanding of the contingencies of tool use. In her opinion, previous tool-use studies could not differentiate whether animals truly understood the parameters of the task or simply learned through trial-and-error which tools are the most effective (including wild chimpanzees that chose nut-cracking tools based on the hardness of the nut) [12].

Although inspecting and switching tools before use was far less frequent than doing so after use in the current study, we did observe a number of occasions when chimpanzees inspected hammers and switched between them prior to using them. This suggests that our subjects attributed a specific function to tool weight, namely that weight is directly related to the effort (via number of strikes and time) required to crack open a nut. Conceivably, the higher prevalence for inspection before use in wild capuchins compared to captive chimpanzees was related to the testing conditions in each study. Recall, that Visalberghi et al. [32] presented capuchins with two hammers differing considerably in weight (639 versus 1820 g) and thus effectiveness. Moreover, the tools were placed at least 3 m to 12.6 m away from the anvils. Under such circumstances the benefits an animal obtains by selecting the appropriate tool in advance is maximized. Thus, it is reasonable that capuchin monkeys inspected hammer weight before starting the costly transportation process. In the current study the weight difference was smaller and all hammers were placed next to the anvil. Consequently, no hammer transportation was necessary.

In a follow-up study, Fragaszy et al. [36] presented the same capuchins with a choice of two stones that differed less in weight (213 versus 572 g) and placed only 50 cm away from the anvil. This considerably reduced the cost of switching from using one stone to another. In contrast to another study [32] where no monkey returned to carry the second stone to the anvil, Fragaszy et al. [36] reported that monkeys switched 19 times (out of 169 trials) from using one stone to another stone in the course of trying to crack a single nut. This switch after initial use indicates that they either applied insufficient search for weight before tool selection or that they had difficulties in recognizing the smaller weight difference.

It is important to emphasize that, even if chimpanzees showed fewer behaviors to gain weight information of the tools in advance, this, by itself, does not indicate a poorer understanding of the functional characteristics of the tool. Previous studies have shown that chimpanzees are able to gain weight information to find hidden food [39] and can infer the location of food based on the effect that its weight has on other objects [40]. In the present study, chimpanzees started to switch tools already before using them, and this behavior became more frequent when the weight differences were increased (Exp. 3). Taken together, we believe that the benefit an animal obtains when choosing a particular tool (measured by the different efficiencies of the presented tools), together with the cost of switching to another tool (measured by the distance between tool and anvil), determines to a large extent tool selectivity.

### Conclusion

Our findings show that chimpanzees actively choose appropriate hammers, based solely on weight, to crack open nuts. Encoding the requirements that a nut-cracking tool should meet (in terms of weight) to be effective therefore lies within chimpanzees’ capabilities. Experience in nut cracking clearly affects subjects’ attentiveness to the tool properties relevant for the task: Loi, our most skilled subject, showed superior performance, and all other individuals improved over the course of the experiments (except for the infant). Studies with wild, skilled and unskilled nut-cracking chimpanzees would help us to better determine the role that experience plays in tool selectivity.
